# ComBatFamQC: Streamlining Interactive Batch-Effect Diagnostics and Harmonization for Neuroimaging Data in R

**DOI:** 10.64898/2026.07.29.741509

**Published:** 2026-08-04

**Authors:** Zheng Ren, Elizabeth A. Horwath, Siyan Wen, Randa Melhem, Jessica K. Anderson, W. Evan Johnson, Russell T. Shinohara, Andrew A. Chen, Haochang Shou

## Abstract

As multisite and multi-study data aggregation becomes increasingly common for improving statistical power and sample diversity, robust harmonization methods are needed to address biases introduced by batch variation, particularly in neuroimaging research. Although a variety of harmonization approaches are available, the lack of systematic guidance for diagnosing batch effects and selecting appropriate methods remains a major challenge. To address this gap, we introduce ComBatFamQC , a comprehensive R package designed to streamline batch-effect diagnosis, harmonization, and post-harmonization analysis. ComBatFamQC integrates a user-friendly Shiny app for interactive batch-effect diagnostics, state-of-the-art harmonization methods from the ComBat family, including ComBat, longitudinal ComBat, ComBat-GAM, and CovBat, and tools for downstream analysis after harmonization. The package provides qualitative visualizations, statistical tests for batch-effect assessment, and a consistent interface that supports both in-sample and out-of-sample harmonization through the Shiny app, the R console, or the command line. In addition, it includes functions for post-harmonization analyses to facilitate downstream modeling. Its modular design also supports the systematic incorporation of future harmonization methods and expanded downstream analysis capabilities.

## Full Text Availability

The license terms selected by the author(s) for this preprint version do not permit archiving in PMC. The full text is available from the preprint server.

